# Chloroform in Hooping Cough

**Published:** 1854-02

**Authors:** 


*Chloroform in Hooping Cough.*—Dr. Churchill recommends chloroform in hooping cough, but thinks it likely to prove of more value in cases of children of twelve years and upwards than in those younger, as they will inhale it more promptly and properly. His mode of exhibition is worthy of attention. "In order to avoid the possibility of an over dose, I have never given the chloroform on a handkerchief or by means of an inhaler, but have directed the mother, (in case of young children,) or the patient, to spill a little, say about thirty drops in the palm of your hand, and hold this before the mouth and nose sufficiently near to inhale it fully, but not so close as to exclude a portion of atmospheric air. The best time to begin is just as the patient feels the irritation in the chest increasing to a cough, but if possible, before the cough commences, and the inhalation should be repeated with each return of irritation, unless headache be produced.—*N. H. Jour. Med.*

